# Postpartum Vertebral Artery Occlusion and Stenosis Following Cesarean Section

**DOI:** 10.7759/cureus.86405

**Published:** 2025-06-20

**Authors:** Hatice S Ercin, Seyma Ozer

**Affiliations:** 1 Family Medicine, Tuzlukcu State Hospital, Konya, TUR; 2 Family Medicine, Umraniye Research and Training Hospital, Istanbul, TUR

**Keywords:** cesarean section, ct angiography, postpartum stroke, spinal anesthesia, vertebral artery occlusion

## Abstract

Vertebral artery occlusion is a condition that can reduce blood flow to the vertebrobasilar system, which may cause various neurological symptoms. We report the case of a 34-year-old female patient who had a cesarean delivery under spinal anesthesia 19 days before presentation. She had no identifiable risk factors. The patient presented to the Family Health Center with complaints of dizziness. She exhibited numbness on the right side of the face, right arm, and right side of the tongue, as well as weakness in her right arm and leg, and was subsequently referred to the emergency department via ambulance. Cranial diffusion MRI showed millimetric diffusion restriction in the right cerebellar and right bulbar regions. Brain CT angiography also showed an occlusion in the V3 and V4 segments of the right vertebral artery. Following medical treatment, the patient’s symptoms improved, and she was discharged with a plan for long-term follow-up. This case underscores the need to consider posterior circulation stroke even in young, low-risk postpartum patients.

## Introduction

Vertebral artery stenosis (VAS) is a condition characterized by significant narrowing of the vessel lumen due to pathological changes in the arterial wall. This stenosis is often seen in the initial segment of the vertebral artery. Stenosis in the vertebral artery has been detected as the underlying cause in approximately one-fifth of ischemic stroke cases occurring in the posterior cerebral circulation [1].

Among the main etiological factors that play a role in the development of VAS are atherosclerotic changes, arterial calcification, vascular wall dissections, fibromuscular dysplasia, giant cell arteritis, neurofibromatosis, and compression of the vertebral artery by surrounding anatomical structures. In cases with clinical symptoms, neurological findings such as visual problems, nystagmus, vertigo, altered consciousness, paresthesia, muscle weakness, nausea, and balance disorders may be observed. In cases that are not diagnosed and treated appropriately, the condition may progress to serious consequences such as ischemic stroke, myocardial infarction, vertebrobasilar circulatory insufficiency, or sudden death. Systemic diseases such as diabetes, hypertension, and hyperlipidemia are among the important predisposing factors for the development of VAS [2].

This case report aims to highlight that vertebral artery occlusion and stenosis in the postpartum period following cesarean section are highly rare. It also emphasizes the importance of considering posterior circulation disorders even in young patients without vascular risk factors.

## Case presentation

A 34-year-old previously healthy female patient presented to the Family Health Center with a complaint of sudden-onset, spinning-type dizziness that began during the night and lasted for several hours without recurrence until presentation. The patient had complaints of numbness in the right half of her face, right arm, and right side of her tongue, weakness in her right arm and leg, and a history of an uncomplicated cesarean delivery under spinal anesthesia 19 days ago. The patient had no known history of chronic illnesses or regular medication use. The physical examination of the patient revealed a fever of 36.5°C, pulse 75/minute, and blood pressure measured in both arms 110/70 mmHg. Peripheral pulses were palpable, and there was no murmur over the common carotid artery on auscultation. Per the neurological examination, the patient's consciousness was clear, she was cooperative and oriented, speech and comprehension were normal, and she had no facial asymmetry. There was no nuchal rigidity. Pupils were isochoric, direct and indirect light reflexes were positive. Eyes were free in four directions, and fundus examination was normal. Other cranial nerve tests were normal. There was no feature in the muscle strength examination. Deep tendon reflexes were normal. Cerebellar tests were competent, tandem walk was competent. She had no sensory deficit, and sitting balance was normal. The patient was deemed to require further examination and treatment due to the severity of her symptoms and was referred from the Family Health Center to the emergency department via ambulance.

The patient's follow-up after this stage was performed via the e-Nabiz system (Turkey’s trusted personal health record system, where the patient and doctors manage health information). Written informed consent was obtained from the patient for publication of this case report and associated images.

**Figure 1:**
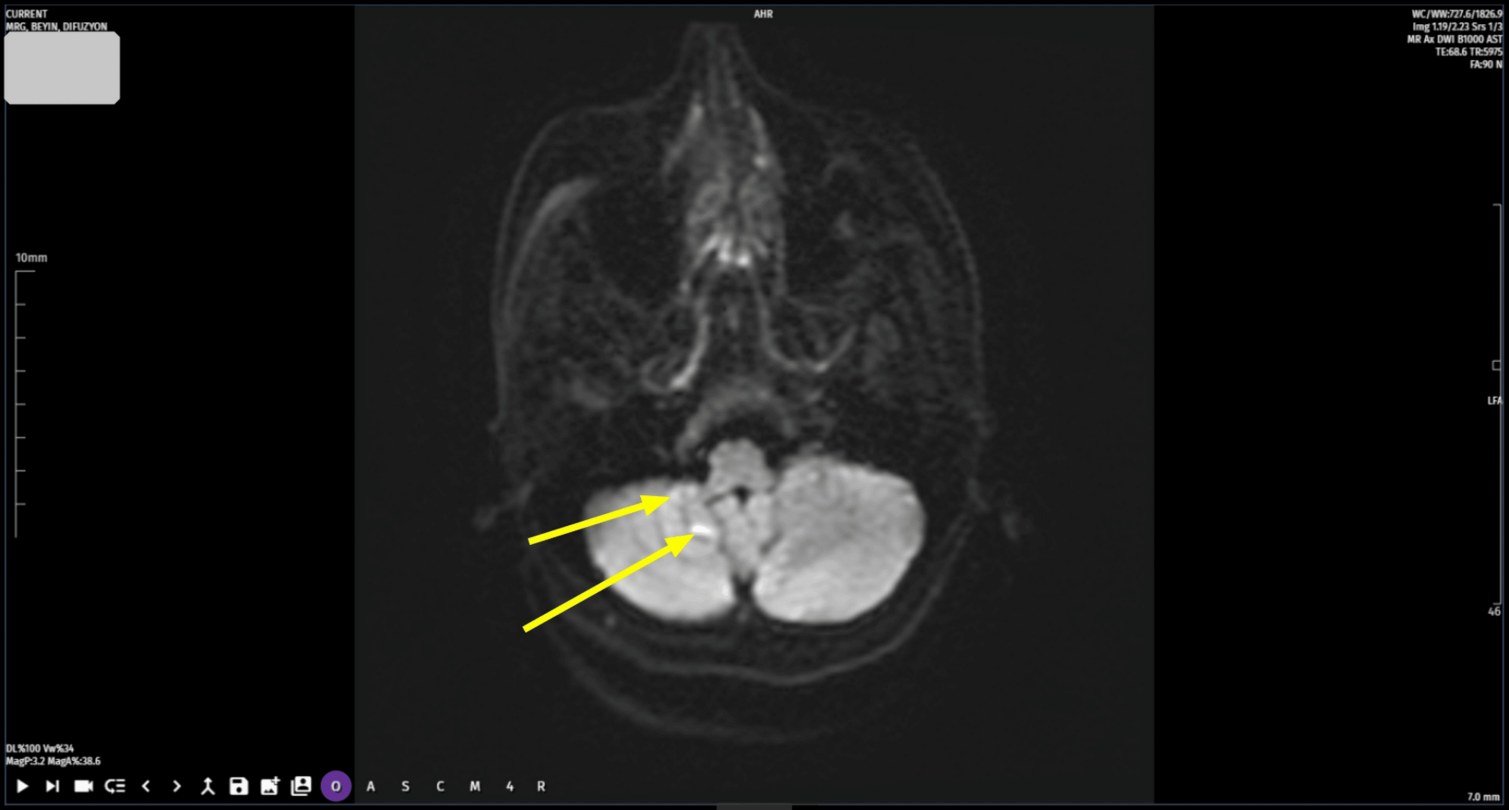
Diffusion-weighted MRI of the brain The arrows point to the hyperintense signals in the right cerebellar hemisphere and bulbar region.

**Figure 2:**
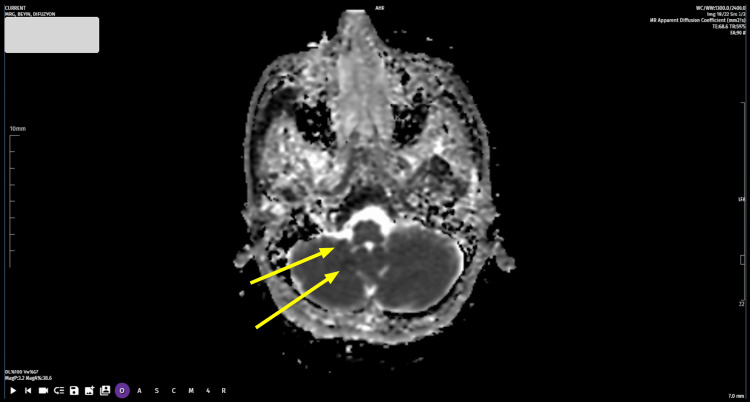
Apparent diffusion coefficient MRI of the brain The arrows point to the hypointense areas, which are consistent with an acute ischemic infarct in the distribution of the right vertebral artery.

The laboratory tests performed in the emergency department revealed no abnormalities in routine hemogram, sedimentation, blood sugar, lipid profile, kidney and liver function tests. The ECG and chest X-ray were normal. Cranial diffusion MRI revealed millimetric diffusion restriction in the right cerebellar area and diffusion restriction in the right bulbar area (Figures 1-2). Brain CT angiography revealed an occluded appearance in the V3 and V4 segments of the right vertebral artery. Following these emergency investigations, the patient was admitted to the neurology department with the diagnosis of vertebral artery occlusion and stenosis.

During the 12-day hospitalization, the patient was started on low molecular weight heparin twice daily, 300 mg/day aspirin (acetylsalicylic acid), and 40 mg/day pantoprazole. Due to occasional nausea, the patient was given ondansetron 4 mg twice daily. A vasculitis and thrombophilia panel was performed with unremarkable results. A Holter monitor revealed no significant findings. After follow-up and treatment, the patient's dizziness and other neurological symptoms decreased, and she was discharged to be followed up in outpatient clinics. The patient was prescribed low molecular weight heparin 6000 anti-Xa IU/0.6 ml/day for six months and lifelong aspirin 300 mg once daily.

## Discussion

Vertebral artery occlusion is most commonly associated with atherosclerosis. However, other factors such as vertebral artery dissection, fibrous bands in the neck, cervical vertebral trauma, and giant cell arteritis may also lead to this condition [3,4].

The diagnosis of etiological causes of posterior circulation symptoms, which range from mild dizziness to dysfunction of the brainstem or occipital cortex (e.g., dizziness, loss of balance, ataxia, tinnitus, and drop attacks), is more complex compared to problems in the anterior circulation [5,6]. This is because the morphological and flow characteristics of the vertebral arteries are often variable and are not as clear and precise compared to carotid examinations.

Understanding these symptoms requires examining the underlying pathophysiology of posterior circulation strokes. The two main pathophysiological mechanisms that are involved in the development of ischemic cerebral infarction are thromboembolism and hypoperfusion. The impact of arterial stenosis on brain tissue depends on the degree of stenosis and the competency of collateral circulation. Stenosis in a single artery has minimal effects on distal hemodynamics unless the luminal narrowing reaches a critical level. However, beyond this critical point, even small increases in stenosis can significantly reduce intravascular pressure and flow. Interestingly, even when angiography shows vessels narrowing between 50% and 75%, cerebral blood flow can remain at normal levels thanks to sufficient collateral circulation [7].

Color Doppler ultrasound (CDUS) is used to determine the flow direction and measure flow volume in the vertebral arteries. However, due to the common occurrence of vascular tortuosity in some cases, assessment of the vertebral artery origin may not always be possible. In such cases, cranial MR angiography and conventional angiography are generally required for diagnosis. Digital subtraction angiography (DSA) not only provides a definitive diagnosis but also plays a critical role in planning endovascular treatment. Today, using conventional angiography or DSA, the length of the stenotic segment and the degree of stenosis can be determined, and if necessary, interventional radiological procedures such as percutaneous transluminal angioplasty or stent placement can be performed in the same session [8-10].

Our case had symptoms that would point to a posterior circulation system disorder, such as dizziness, numbness in the face, arm, and tongue, and weakness in the arm and leg. No abnormalities were detected in the physical examination and laboratory tests of our patient. Although she had no known vascular risk factors, due to the presence of neurological symptoms, a brain CT angiography was performed to elucidate the etiology, and an occluded appearance in the V3 and V4 segments of the right vertebral artery was revealed.

On scanning the literature for pregnancy-related vertebral artery pathologies, only a limited number of case reports are encountered. The majority of these are vertebral artery dissections, with none on the association between vertebral artery stenosis and pregnancy. The risk is notably higher during the peripartum and postpartum periods than the pregnancy period, particularly in women with hypertensive disorders of pregnancy [11,12].

It is suggested that the risk of stroke resulting from vertebral artery stenosis can be reduced by the use of antithrombotic and anticoagulant drugs [13,14]. Surgical intervention has been abandoned as a routine practice due to the frequency of complications such as Horner syndrome or laryngeal nerve damage. Balloon angioplasty and stent placement procedures can also be performed with medical treatment, but no comparative studies have shown the effectiveness of these methods. This situation shows that the treatment of VA stenosis does not have as clear boundaries as the indications for the treatment of anterior system circulation problems. For these reasons, we preferred to choose medical treatment instead of surgical intervention for our patient. But during medical treatment, it was taken into account that our patient had just given birth and was keen on avoiding using medication as she wanted to breastfeed. So we chose medications that are suitable for breastfeeding. Measures were taken to address potential risk factors, and follow-up treatment with low molecular weight heparin was planned.

## Conclusions

Regardless of whether they have vascular risk factors, it's essential to consider a wide range of possible causes when postpartum patients present with neurological symptoms such as dizziness. Even in the absence of common vascular risk factors, serious conditions like vertebral artery occlusion can occur. From a primary care and family medicine perspective, early identification, appropriate referral, and coordination of care are all key to helping such patients. Family physicians, who are often the first point of contact, play an indispensable role in recognizing red-flag symptoms, considering rare but serious causes, and ensuring continuity of care during the vulnerable postpartum period.
